# HealthLit4Kids: teacher experiences of health literacy professional development in an Australian primary school setting

**DOI:** 10.1093/heapro/daac053

**Published:** 2022-05-11

**Authors:** Claire Otten, Rose Nash, Kira Patterson

**Affiliations:** 1 School of Medicine, College of Health and Medicine, University of Tasmania, Private Bag 34, Medical Science 2 17 Liverpool Street, Hobart, TAS 7001, Australia; 2 School of Education, College of Arts, Law, and Education, University of Tasmania, Locked Bag 1345, Launceston, TASMANIA 7250, Australia

**Keywords:** health literacy, children, education, school, prevention

## Abstract

Health literacy (HL) is a critical asset for 21st century learners to possess given its positive impact on health outcomes and educational attainment. Concerningly, HL is an area that primary school teachers report having a lack of understanding, confidence, and ability to teach. The HealthLit4Kids initiative aimed to address this issue through a series of teacher professional development (PD) workshops. To evaluate how teachers experienced the PD, teacher evaluations collected at the completion of each of the workshops were analysed using a mixed methods approach. According to the teachers, the PD had improved their understanding of HL, including how to implement it into their practice. The study also found that the teachers perceived that collaborative practice was a key strength of the programme, and that at the end of the PD, teachers described valuing HL more in their practice. Teachers reported time as a major barrier to them implementing the professional learning and suggested further resources could help to mitigate this barrier. Additional research is required to help substantiate the claims made in this research. This study also highlights the critical need for additional HL resources for primary school teachers.

## INTRODUCTION

Due to the growing complexity of many health systems globally, the skills required to engage with health services and health information are increasing [Australian Institute of Health and Welfare (AIHW), 2018]. The ability to navigate health information is a key determinant of health [World Health Organisation (WHO), 2010]. Health literacy (HL) impacts an individual’s ability to understand health information and navigate health systems, and consequently, their capacity to form educated and autonomous health-related decisions (Okan *et al.*, 2019). HL can be defined as

the ability to make sound health decision[s] in the context of everyday life—at home, in the community, at the workplace, the healthcare system, the marketplace and the political arena. It is a critical empowerment strategy to increase people’s control over their health, their ability to seek out information and their ability to take responsibility [(Kickbusch *et al.*, 2005), p. 4].

HL is, therefore, a critically important collective priority.

The need for HL promotion in schools has been widely acknowledged (Paakkari and Okan, 2019). Primary schools specifically have been recognized as key settings to develop children’s HL (Nutbeam, 2000; Leger, 2001; Paakkari and Paakkari, 2012). This is because childhood is an important time to develop an individual’s HL (Velardo and Drummond, 2017; Bröder and Carvalho, 2019) and HL has been shown to improve through informal and formal education (Nutbeam and Mcgill, 2019). Consequently, teachers must be equipped with appropriate skills and resources, to ensure that primary schools, can support the development of HL for all children and their communities.

Research indicates that many teachers do not feel competent or have the adequate professional knowledge required to teach health (Lynagh *et al.*, 2010; Petrie *et al.*, 2014). Professional knowledge can be divided into three sub-categories; content knowledge (CK), pedagogical content knowledge (PCK) and pedagogical knowledge (PK) (Shulman, 1986). CK refers to an understanding of the topic being taught, PCK to an understanding of how to teach that specific topic, and PK to general teaching practices that facilitate effective learning environments (Kulgemeyer and Riese, 2018).

In Australia, health is often taught by the classroom teacher, rather than a health and physical education specialist (Lynch, 2013). Unlike health and physical education specialists, classroom teachers are responsible for teaching multiple subjects and spend the greatest number of hours with students. Although primary teacher education courses in Australia include health education as a topic during the degree, health pedagogy receives little attention when compared to health and physical education specialist courses (Cruickshank *et al.*, 2021). As teachers are more likely to teach content areas that they are familiar with (Barwood *et al.*, 2017), health may be an undertaught area of the curriculum. This justifies the need for ongoing teacher education, which can be achieved through professional development (PD) (Zepeda, 2012).

The HealthLit4Kids initiative addresses this problem through a school-based HL intervention (Nash *et al.*, 2018). HealthLit4Kids is a programme designed to improve the HL of children and their school community. Over 12 months, three interactive PD workshops are delivered in the school setting to teachers. The workshops include individual, small group and large group activities that aim to develop teachers understanding of what HL is, as well as how to promote HL development for their students.

The aim of the current study is to evaluate the teacher experience of the HealthLit4Kids PD programme. Specifically, to determine programme acceptance, and if there was a perceived change in teacher knowledge and confidence to teach HL following the PD workshop series. To meet this objective, the following research question was posed:

How did teachers at three Tasmanian primary schools experience the HealthLit4Kids (HL) PD program?

## METHODS

### Programme background

HealthLit4Kids responds to a gap in the delivery of HL programmes to communities and schools by working at a local level with children, schools, families and communities to develop new approaches to learning and health (Nash *et al.*, 2018). During the three PD workshops, teachers were invited to engage in a range of activities including: learning about HL and its application in an educational context, undertaking a survey to determine their baseline HL, defining what a health literate school looks like, assessing the HL responsiveness of their school, learning how to implement a school action plan at a classroom and whole of school level and planning an expo to showcase student learning. Teachers were encouraged to select a HL focus that was relevant to their students (e.g., nutrition).

Given that HealthLit4Kids is a whole of school and classroom level intervention, multiple activities and data were collected throughout each PD workshop. The data serves three purposes: educative for teachers (HL pedagogy and CK), school specific action plan development and data collection for programme evaluation. Teachers were invited to self-assess their own HL knowledge skills and experience, to assess their school’s organizational HL responsiveness (Elmer *et al.*, 2021), and set a whole of school action plan (with short- and long-term goals). Teachers also had opportunities to develop lesson plans to practice embedding their knowledge of HL into the classroom aligned with their school’s goals. The final workshop encouraged teachers to share their insights from the programme and solidify their understanding of individual HL development, HL responsiveness and distributed HL in their community. This article reports on teacher evaluations of the PD workshops. Please refer to following references for other research findings from the programme (Nash *et al.*, 2018, 2019, 2020, 2021a; Elmer *et al.*, 2021).

### Methodological framework

HealthLit4Kids adopts a duel transformative and pragmatist philosophical worldview (Creswell, 2013). A multi-phased mixed methods approach was employed; involving the integration of both qualitative and quantitative data (Teddlie and Tashakkori, 2009). This study combined convergent parallel mixed methods, with sequential mixed methods (Greenhalgh *et al.*, 2016). As this study attempts to explain the impact of PD on teachers over a series of three PD workshops, a sequential approach was employed. To strengthen the study, there was convergence of data at each separate time point (outlined in Supplementary Figure S1).

### Participants

The participants in this study were the teachers who were involved in the PD workshops. Schools were chosen to be involved in the HealthLit4Kids programme based on their accessibility and willingness to adopt the programme (Supplementary Table S1). Whilst five schools participated in the programme, this research will present the findings from three schools only. All three schools were located in Tasmania, Australia. The same facilitator led the PD in the three schools from which data were collected. A total of 113 responses were collected over three time points from 48 individual teachers (Table 1). Incomplete data from two of the participating schools made it difficult to include them in the sub-study presented here.

**Table 1:** Mixed data analysis

Questionnaire question	Research sub-question	Qualitized quantitative findings	Qualitative response		Combined finding
			Response/key findings	Examples	
1a. Where your expectations met in terms of a clear definition of HL with adequate knowledge and understanding to enable application in your own situation? **(yes/no/unsure)**	1. Did the HealthLit4Kids PD meet the teacher’s expectations in terms of a clear definition of HL?	- Post HealthLit4Kids PD most teachers reported having a clear understanding of a how HL can be defined, as well as how to implement it into their lessons - No teachers reported the programme not providing them with a clear definition of HL with inadequate knowledge and understanding to enable application in their own setting - Most teachers understanding of HL developed throughout the series of workshops	- Almost all teachers reported an understanding or the development of understanding of the term HL	‘The workshop helped me understand what encompasses health literacy’. ‘The definition and expectations of HealthLit4Kids of health literacy were clear’. ‘I now have a clear understanding of what health literacy means’. ‘I have a clearer understanding of health literacy after the session’. ‘In the beginning I only had an “educated guess” as to what health literacy was. Now I’m not “guessing”’.	- Quantitative and qualitative responses were congruent with one another - The HealthLit4Kids PD met teachers’ expectations in terms of providing a clear definition of HL
	2. Did the HealthLit4Kids PD meet the teacher’s expectations in enabling them to implement HL in their classroom lessons? If so, how?		- Teachers reported an increased ability to incorporate HL into their classroom lessons at the end of the PD	“I’m now able to assist parents with information. Highlight ‘health’ related areas. Plus feel confident teaching health.” “Consolidated my understanding of health literacy- and how I can apply it.” “I expected to find out what health literacy is. The workshop exceeded my expectations—inspired us to think about how we can implement.”	- Quantitative and qualitative responses were congruent with one another - The HealthLit4Kids PD met teachers expectations in terms of enabling them to implement HL in their classroom lessons
1b. Where your expectations met in terms of understanding the elements that influence the HL of the school environment and ideas on how to use them in your own situation? **(yes/no/unsure)**	3. Did the HealthLit4Kids PD lead to a development in understanding the elements that influence the HL of the school environment?	- The PD met the teacher’s expectations in terms of understanding the elements that influence the HL of the school environment and ideas on how to use them in their own situation - A greater number of teachers reported positive responses in each subsequent workshop	- Overall, teachers reported an understanding of the elements that influence the HL of the school environment at the end of the HealthLit4Kids PD	‘I hadn’t thought a lot about the “big picture” in terms of health literacy in my present position at this school. I’m now more aware of what it might mean for us’. ‘[The PD] gave an excellent understanding of all the elements involved in health literacy and how they apply to all areas of the school community’. ‘The workshops have enabled us to undertake conversations at a whole of teaching staff level that have supported us in developing our collective understanding of the elements that influence the health literacy of the school environment’. ‘School wide focus as well as class focus for health literacy was clearly outlined’. ‘We have a shared definition of what health literacy is and why it is important’.	- Quantitative and qualitative responses were congruent with one another - The HealthLit4Kids PD met the teacher’s expectations in terms of understanding the elements that influence the HL of the school environment
	4. Did the HealthLit4Kids PD enable teachers to implement HL in the school environment?		- Although the written responses in the questionnaire that were made in relation to teachers developed knowledge of how to implement HL in the school environment were positive, there was not enough evidence to support of make any claims	*What was useful about the workshops?* ‘All staff working together to create a whole school approach to being a healthy lit school’. ‘To develop a whole school approach together as a team’. ‘Gave an excellent understanding of all the elements involved in health literacy and how they apply to all areas of the school community’.	- Quantitative and qualitative responses were congruent with one another - The HealthLit4Kids PD met the teacher’s expectations in terms of enabling teachers to implement HL in the school environment - This may be an area for future consideration, as overall the qualitative responses lacked depth
2. Do you agree with the HL focus areas identified by/for your school? **(yes/no/unsure)**	5. Did the HealthLit4Kids PD outline HL focus areas that teachers considered relevant to the needs of their school?	- Teachers reported an increase in agreeance of the HL focus areas in their school throughout the PD programme - Initially, just over half of the teachers reported an agreeance - Almost all teachers agreed with the focus areas by workshop three - No teachers did not agree with the focus areas	- Many teachers reported being unaware of what the specific HL focus areas were for their school - The responses from questions across the survey, indicated that teachers were happy HL was a general target for their school	‘Good to be able to target areas identified by staff as relevant to our school community’. ‘The workshops have enabled us to undertake conversations at a whole of teaching staff level that have supported us in developing our collective understanding of the elements that influence the health literacy of the school environment’. *What was useful about the workshops?* ‘All staff working together to create a whole school approach to being a healthy lit school’.	- By the end of the PD programme, the majority of teachers agreed with HL as a general focus for their school - Many teachers reported initially being unsure of the specific HL focus areas for their school
3. What was useful about the workshop?	6. What elements of the HealthLit4Kids PD did the teachers find useful?	- Not applicable as quantitative data were purposefully not collected in relation to this question	- The workshop helped teachers to recognize the needs or give teachers ideas on how HL could be implemented in their setting - The workshop helped teachers to understand/clarify the meaning of HL and/or understand HealthLit4Kids - Teachers found the collaboratively learning about HL useful - Teachers found the chance to reflect a useful part of the PD	*The most useful element of the HL PD was:* ‘Finding out what health literacy is and how it’s relevant to us’. ‘Sharing ideas and so being able to get ideas for what we could do in class’. ‘A few practical ideas and suggestions for improvement discussed’. ‘Having a whole of staff discussion and realising different perspectives and what is important to different people’. ‘[The workshop] increased my understanding and gave planning/reflection time’.	- Not applicable as quantitative data was purposefully not collected in relation to this question
4. How will you use this information?	7. How will teachers use the information conveyed during the HealthLit4Kids workshop?	- Not applicable as quantitative data were purposefully not collected in relation to this question	- Teachers reported planning on using information from the HealthLit4Kids PD to inform classroom teaching - Teachers reported planning on using information from the HealthLit4Kids PD to implement HL in broader the broader school community	*Teachers reported planning on using the information conveyed during the HL PD:* ‘To inform my teaching practice’. ‘Design lessons and cross curricular activities’. ‘Able to apply this directly to classroom work and school goals moving forward’. ‘To direct future planning in the school and in or own classes’. ‘Integrate into my family partnership model training to support families’.	- Not applicable as quantitative data was purposefully not collected in relation to this question
5. Are there any organizational or other barriers to you using this information? **(yes/no/unsure)**	8. Did teachers involved in the HealthLit4Kids PD report barriers existing that prevented them from implementing HL into education? If so, what were these barriers?	- Results were heterogeneous - Some teachers reported their being organizational/other barriers to them implementing the information learnt during the HL PD, others reported no barriers existing - Just under half reported being either unsure or did not answer the question	- Barriers that teachers reported were lack of parental involvement/engagement, unfamiliar terminology, time, access to resources and department support	*Barriers to using the information from the PD are:* ‘Potentially some parents will be resilient to this information due to their own beliefs’. ‘Accessing new info/resources’. ‘Some terminology that we don’t use’. ‘Incorporating into full curriculum’. ‘Education Department needs to be on board. Education Department needs to find $$’.	- Quantitative responses neither supported, nor negated the qualitative findings. - Responses indicate that not all teachers report their being barriers to them implementing what they learnt in the HL PD; however, those who did found that the barriers were lack of parental involvement/engagement, unfamiliar terminology, time, access to resources and department support
6. What could help remove those barriers?	9. What do teachers believe could help to overcome barriers that prevented them from implementing HL into education?	- Not applicable as quantitative data were purposefully not collected in relation to this question	- Teachers reported department/whole of school approach and support, communication, further PD and access to resources, more time spent on HL and more opportunity for discussion/sharing, and parental support as methods of overcoming barriers to them implementing information learnt during the HL PD	*Factors that could help to remove barriers to implementing HL are:* ‘Community involvement, Department Support, leadership on board’. ‘Communication—learning visible on a seesaw’. ‘Another workshop to help us get things started, so everyone feels confident to move forward/act on things in the class’. ‘Careful communication through a variety of avenues, embedding health lit into the school’. ‘Parent support and more unpacking’.	- Not applicable as quantitative data were purposefully not collected in relation to this question
7. Any suggestions for changes or improvements to the workshop/project? **(yes/no/unsure)**	10. Did teachers report the HealthLit4Kids PD requiring any changes/improvements? If so, what were these changes?	- Slightly more than half of the teachers reported either being unsure or did not answer if they had suggestions for changes of improvements to the workshops - The majority of teachers who selected a yes/no response suggested that the workshop required no altering - A small number of teachers indicated that elements of the workshop would be benefit from altercation	- A large number of teachers reported the workshop requiring no altercation - Some reported a greater amount of time to spend on the PD and more opportunity for discussion/sharing as factors that could have further strengthened the PD	*Changes that could be made to the HL PD are:* ‘None it’s great’. ‘None very happy’. ‘More time. I know this is always a tough one, but it would allow to build knowledge of students’. ‘More sharing between classes. More time to deeply engage children before the showcase. Showcase the process not just the artefact’. ‘An opportunity for students and teachers to share their learning and artefacts’.	- Quantitative and qualitative responses were congruent with one another - Most teachers did not believe that the HL PD required altercation - The small number of teachers who reported the PD requiring changes, suggested that a greater amount of time to spend on the PD and more opportunity for discussion/sharing were factors that could have further strengthened the PD

### Data instrument/collection

The data were collected using evaluation questionnaires. A workshop evaluation questionnaire was created by the HealthLit4Kids research team, to assess the effectiveness of the programme. As there is a paucity of HL PD programmes for teachers delivered in primary school contexts internationally (Nash *et al.*, 2021b; Otten *et al.*, 2022), there is also a lack of tools to measure programme effectiveness. Consistent with a pragmatist approach, a tailored evaluation technique was developed (Creswell, 2013). The questionnaire used for this research was comprised of eight questions. Thirteen response were required to complete the questionnaire. Three questions required an open-ended response and five questions required an open-ended response and a trichotomous response (i.e. yes/no/unsure) (Supplementary Figure S2). All questions were in relation to teacher experience of the HealthLit4Kids PD. At the completion of each workshop, researchers invited teachers to complete the questionnaire. To reduce participant/response bias, a number of strategies were implemented including making all survey responses anonymous, as well leaving a box for participants to place their survey into rather than the researchers directly collecting the surveys.

### Data analysis

As this study adopted a multi-phased mixed methods approach, data were analysed using a variety of methods (Creswell, 2013; Greenhalgh *et al.*, 2016). High quality mixed methods are more likely to embed integration throughout the study (Greenhalgh *et al.*, 2016). Parallel mixed data analysis where qualitative and quantitative data is analysed independently, and then compared to create meta-inferences was employed (Creswell, 2013). This was useful as the strengths of one design could be used to overcome the limitations of another (Greenhalgh *et al.*, 2016). Conversion mixed data analysis involving the transformation of one data type to another (i.e. quantitative to qualitative) was also used (Teddlie and Tashakkori, 2009). Findings from one form of data can be compared to another, thus, strengthening the results of the study (Greenhalgh *et al.*, 2016).

Descriptive statistics was used to analyse the quantitative data. The trichotomous questions from the questionnaire provided categorical/nominal data (Greenhalgh *et al.*, 2016; O’Leary, 2017). In this study, quantitative questionnaire questions were first coded (1 = yes, 2 = no, 3 = unsure). Frequencies were determined using percentages (Walliman, 2017). As the research aimed to explore the teachers experience (dependent variable) of a HL PD (independent variable), at several time points (continuous variable), a bi-variance approach (considering how pairs of questions interact or are different) was employed (O’Leary, 2017; Walliman, 2017). This data was displayed using clustered bar charts (O’Leary, 2017).

Reflexive thematic analysis was used to analyse our qualitative data (Braun and Clarke, 2021). Due to the gap within the existing literature (Nash *et al.*, 2021b; Otten *et al.*, 2022), no relevant framework exists for assessment of HL PD in a primary school setting. As such, analysis of questionnaire responses was not informed by prior literature but was instead inductively assessed and themes derived.

Though inductive in assessment, a deductive element exists within this qualitative analysis. As data were derived from responses to open-ended questions, we acknowledge that there is the potential for participant responses to be led towards the inclusion of language via the question itself. For example, a question pertaining to knowledge ‘Were your expectations met in terms of a clear definition of HL and adequate knowledge and understanding to enable application in your own setting’, is likely to evoke participants to discuss their HL knowledge. Similarly, a deductive element (subjectivity) was introduced into the analysis phase via the researcher searching for themes that relate to the language and the underlying premise of the question. This subjectivity is a key principle of reflexive thematic analysis that allows researchers to inductively retrieve themes from the data without the risk of deriving patterns that do not pertain to the outcome (question) of interest (Campbell *et al.*, 2021).

Five of the eight questions in the questionnaire incorporated both qualitative and quantitative data (see Supplementary Figure S2). Quantitative data relating to these five questions were qualified (qualitization) (Tashakkori and Teddlie, 1998). This was through the interpretation and transcription of ordinal data into qualitative written findings. This is useful as it extracts further information from the data set (Sandelowski, 2000). Further, data transformation allows for the convergence of both data types into a single data set (Greenhalgh *et al.*, 2016). This study compared both the qualitative and qualitized quantitative data according to each question in the questionnaire. Responses were then compared to determine degree of congruency (Table 1).

## RESULTS

Results are presented in three sections. The first two sections present the quantitative and qualitative data respectfully, as separate entities. The final section presents converged mixed data.

### Quantitative results

**Fig. 1:**
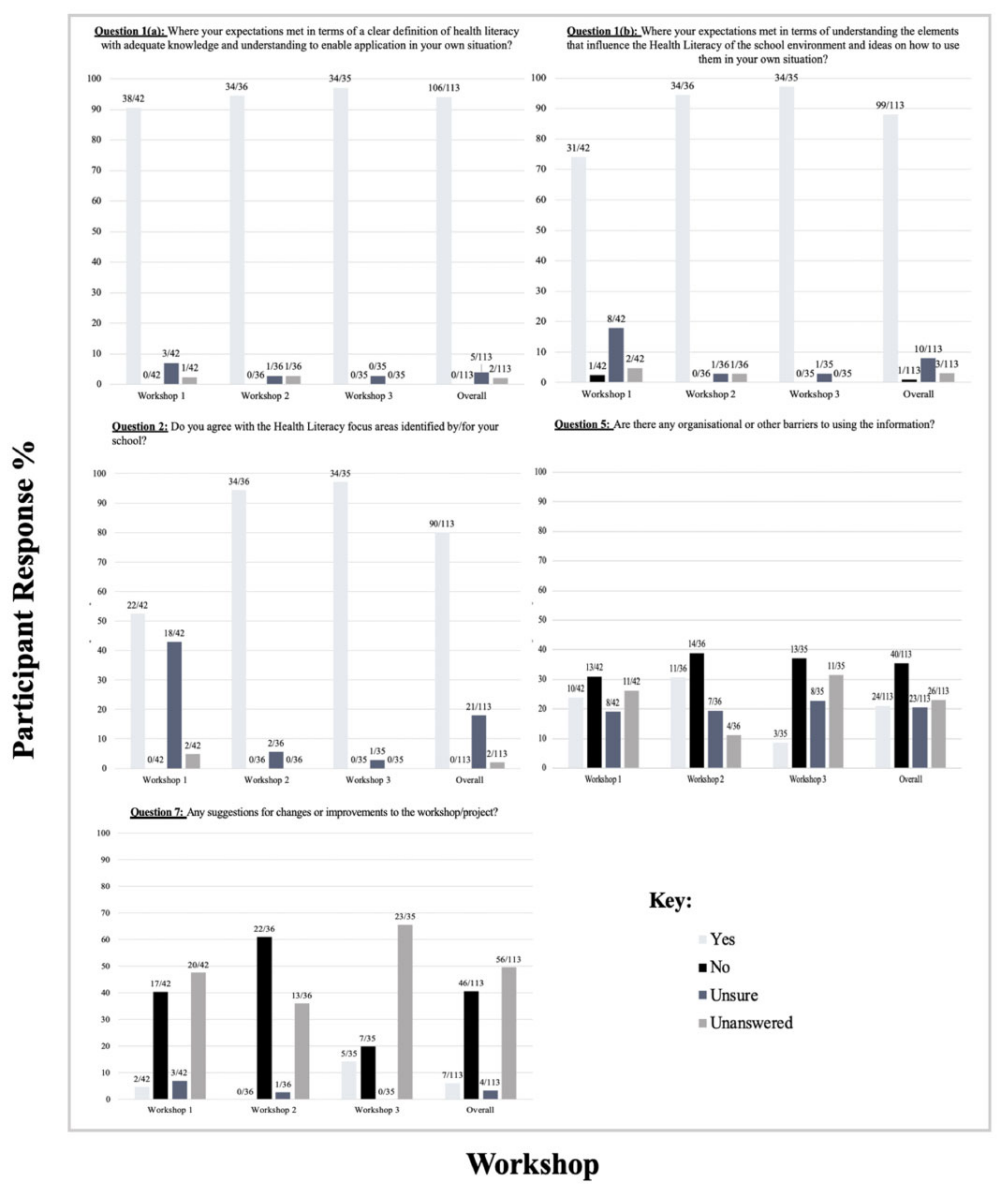
Quantitative data analysis.

The data from schools A, B and C have been grouped. The results in Figure 1 present the findings from the quantitative component of the questionnaire. The findings demonstrate that overall 93.8% of teachers reported that the HL PD provided a clear definition of HL with adequate knowledge and understanding to enable application in their situation. No teachers reported it not meeting their expectations. There was a gradual increase of 6.6%, with 90.5% of participants suggesting that the PD provided a clear definition, in contrast to 97.1% selecting ‘yes’ in workshop three. From workshop one to workshop three, there was also an increase of 22.3% in teacher’s expectations being met in relation to understanding the elements that influence the school environment and ideas on how to use this in their own situation. Throughout the PD, 79.7% of participants reported agreeing with the HL focus areas for their school, with no teachers reported not agreeing. Across the three workshops, 35.4% of participants suggested that there were not any barriers to them implementing the information, 21.4% reported that there were, 20.4% responded that they were unsure and 23% did not answer the question. Of the 57 teachers who responded 81% suggested that the workshop did not require any changes, 12.3% reported that it did and 7% reported being unsure.

### Qualitative results

**Fig. 2:**
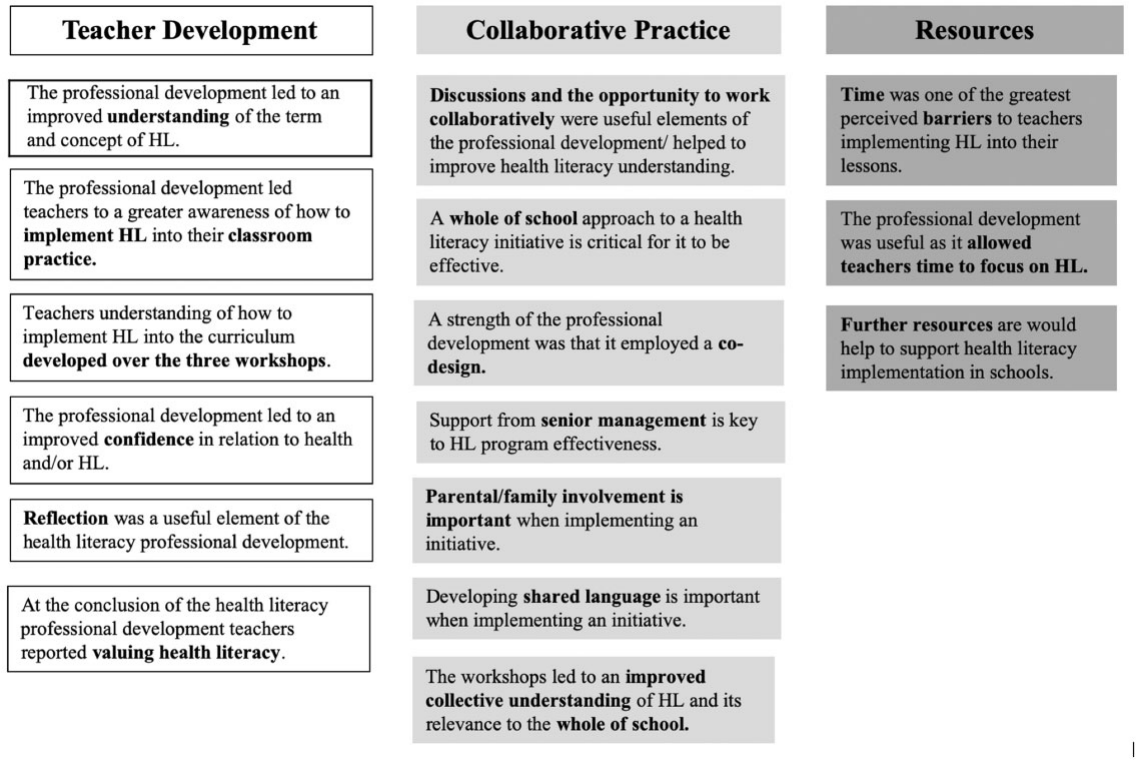
Qualitative data analysis, HealthLit4Kids PD teacher reflection themes.

A number of parent, as well as sub-themes emerged from the analysis. These related to teacher development, collaborative practice and the availability of resources (Figure 2 and Supplementary Table S3). The theme that emerged from the data the most frequently was teacher development. Results suggested that the HealthLit4Kids PD led to a perceived improved understanding of HL. For example, ‘I have a **clearer understanding** of health literacy **after the session’** (Teacher 4, Workshop 1). In addition, the PD led to an improved confidence in understanding, ‘After attending the workshop, **I am now confident** that I understand the term’ (Teacher 2, Workshop 1). This was developed across the three workshops. Collaborative practice was another key emergent theme. Teachers indicated that discussions/the opportunity to collaborate was useful in developing a collective understanding of HL. ‘The **workshops have enabled us to undertake conversations** at a whole of teaching staff level that **have supported us in developing our collective understanding** of the elements that influence the HL of the school environment’ (Teacher 16, Workshop 2). There was a high congruency of themes between participating schools. The same parent themes emerged from the varying data sets. Although there were minor differences in sub-themes amongst schools, none of the data suggested discrepancies or contradictions between sites.

### Mixed data analysis

Table 1 reports the findings from the merged qualitative and qualitized quantitative data based on the 10 research sub-questions. Findings demonstrate that there was a high degree of consistency between the two data types, with six of the seven comparisons supporting one another. One of the seven comparisons found that the qualitized quantitative data neither supported nor negated the qualitative findings (‘Question 5: Are there any organizational or other barriers to you using this information?’).

Teacher’s expectations were met in terms of the programme providing them with a clear definition of HL, which enabled them to implement HL in their classroom. Their newfound understanding of the elements that influence the HL of the school environment enabled them to implement this information. Although many teachers reported being initially unsure of what the HL focus areas for their school were, they agreed that HL as a general concept should be a school target area.

Many participants were unsure if there were barriers that prevented them from implementing HL (Figure 1). Qualitative findings supported this, with many who answered unsure leaving the written component of this question unanswered or responding with ‘unsure’. Those who reported barriers suggested that these were lack of parental involvement/engagement, unfamiliar terminology, time, access to resources and inadequate department support. Many participants reported the PD required no major changes to its design. Those who suggested it did reported that a greater amount of time to spend on the PD and more opportunities for discussion/sharing could have further strengthened the programme.

## DISCUSSION

This study aimed to reveal how teachers at three Tasmanian Primary schools experienced the HealthLit4Kids PD. Evaluations demonstrated that the workshops led to a perceived improvement in teacher’s professional knowledge/understanding of HL, that collaborative practice and reflection were key strengths of the programme and that this resulted in an increase in the value that teachers placed on education that aimed to develop HL. Participants reported that resources were a major barrier to them implementing the professional learning. Many of these findings are supported by other evaluative research on the programme (Supplementary Table S2).

The workshop evaluation findings will now be discussed by theme: teacher development, collaborative practice and availability of resources.

### Teacher development

#### Professional knowledge

The HealthLit4Kids PD led to teachers reporting an improved professional knowledge of HL. This is important given knowledge is a key component of effective teaching (Fischer *et al.*, 2012, 2014). As a result of engaging in the HealthLit4Kids programme, teachers suggested an increase in their HL CK and PCK and were provided opportunities to apply their PK to their teaching context.

An increase CK was reflected in participants’ reported improvement in their understanding of the term HL throughout the workshop series. Several teachers indicated that they did not understand the term prior to the workshops. For example, ‘In the beginning I only had an “educated guess” as to what HL was. Now I’m not “guessing”’. As perceived understanding is linked to confidence (Bandura, 1977), this may explain why many teachers have previously described not feeling confident to teach health-related topics, despite it being an area of the curriculum that they are expected to teach (Deal *et al.*, 2010; Hivner *et al.*, 2019). Although not all teachers understood the term initially, almost all (97.1%) participants reported having a clear understanding of the term HL by the final workshop. This is consistent with prior research that suggests that PD strengthens teacher’s knowledge of health concepts/behaviours (Nash *et al.*, 2021b). Further, it is supportive of the notion that engagement with the HealthLit4Kids programme leads to an improved understanding of HL (Nash *et al.*, 2020, 2021a; Elmer *et al.*, 2021) and that PD should occur over an extended period of time (Elmer *et al.*, 2021; Nash *et al.*, 2021a).

Findings of this study support the suggestion that a relationship exists between CK and PCK (Hill *et al.*, 2005). Consistent with other evaluative research on HealthLi4Kids many teachers reported that the PD led to a greater awareness of how to implement HL into their classroom practice, and in their broader school environment (PCK) (Nash *et al.*, 2021a). This finding was supported through student artefacts, lesson plans and the school expo. A combined CK and PCK has been shown to increase quality of instruction (Hill *et al.*, 2005). Further, a positive correlation has been found to exist between PCK and student learning outcomes (Ergönenç *et al.*, 2014). HealthLit4Kids, therefore, has the potential to lead to positive learning outcomes, through improved teacher CK and PCK.

Educators with strong PK employ contextually appropriate teaching strategies, thereby, facilitating student-centred teaching (Killen, 2015). As student-centred teaching is strongly correlated with positive learning outcomes (Bara and Xhomara, 2020), it is critical that teachers employ their PK to their practice. Further, teachers who adapt programmes to suit the needs of their context, may demonstrate better engagement with PD than those who are asked to implement rigid instructional programmes. This was reflected in this study, with teachers reporting the co-designed approach a strength of the initiative. It allowed them to determine the HL content, and the method of delivery most appropriate to their context and, therefore, exercise their PK.

**Fig. 3:**
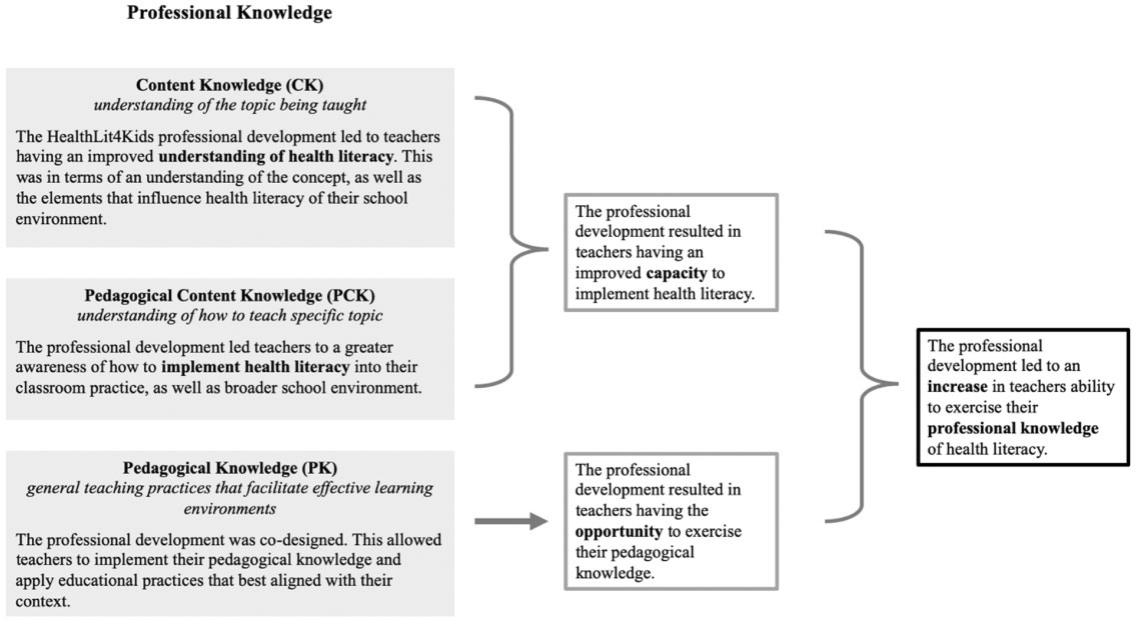
HealthLit4Kids Impact on Professional Knowledge.

Increasing teacher’s professional knowledge of HL could have led to an improvement in student understanding of HL (Figure 3). This may explain why a range of data associated with the programme (i.e., parent interviews and teacher written reflections) indicated that the initiative led to an improvement in student understanding of HL and observed increase in engagement with health promoting behaviours (Nash *et al.*, 2019, 2020, 2021a).

#### Reflection

Teachers at all three schools reported that reflection was a strength of the PD programme. Their responses demonstrated that both individual reflection opportunities, as well as shared reflection time was useful. Reflection has been shown to reinforce learning and can help to provide meaning to experiences (Boud *et al.*, 1985; Hattie, 2009; Ribeiro *et al.*, 2019). This may also help to explain why teachers demonstrated an improvement in their understanding of HL. Collectively reflection (Høyrup and Elkjær, 2006) in the workshops allowed teachers to share their ideas with others teaching in a similar context. As a result, it may have led to an improved understanding of HL, and lessons tailored to be contextually relevant to the needs of their students.

#### Value

Teachers reported valuing HL at the end of the PD, despite many teachers being unaware of the concept prior to the workshops. For example, ‘The HealthLit4Kids concept is excellent and well needed given the current health issues in the community’. Further, they indicated valuing HL education: ‘I believe exposing children to the concept of HL is very important, needs to be ongoing and really embedded across our whole school!’ Many teachers reported being unaware of the concept of HL prior to the workshops, thus, it could be suggested that engagement in the programme led to an appreciation for the importance of education that supports HL development. The significance of these findings cannot be understated, as teachers are more likely to teach concepts that they consider valuable (Kallemeyn, 2019). This implies that their engagement with the PD increased the likelihood of teachers incorporating HL into their lessons. Importantly, teachers play a pivotal role in the development of student values (Kaur and Nagpal, 2013). This is because education impacts one’s worldview, and therefore, what they perceive as important (Narvaez and Lapsley, 2008). Values can be taught implicitly and explicitly (Muthigani, 2019). Consequently, implementing HL into various areas of the curriculum has the potential to implicitly demonstrate to students the value of HL.

### Collaborative practice

A strength of the HealthLit4Kids programme is that it provided opportunities for collaboration through co-design, allowing time for discussion, and supporting the involvement of the broader community. Like other evaluations of the programme (Nash *et al.*, 2020, 2021a; Elmer *et al.*, 2021) teachers reported that the co-design approach embedded in the HealthLit4Kids programme facilitated the creation of lessons that were relevant to the needs of their students; a critical element of effective teaching (Glasersfeld, 1995; Killen, 2015). Bröder *et al.* (Bröder *et al.*, 2020) have highlighted that education designed to promote HL in young people must be contextual, tailored and holistic. Teacher evaluations also revealed that frequent opportunity for discussion was a useful element of the workshops. This is unsurprising, given that it has already been identified as a strength of the programme and collective learning has been evidenced as key to effective PD (Elmer *et al.*, 2021; Nash *et al.*, 2021a). Discussions provide time to plan collaboratively. Collaborative planning encourages innovative practice, with real world problem solving, as a result leading to an improvement in teachers’ CK, as well as PCK (Koh *et al.*, 2017). Further, as suggested in other published HealthLit4Kids findings (Nash *et al.*, 2019, 2020, 2021a; Elmer *et al.*, 2021), teachers acknowledged the importance of employing a whole of school approach. This is significant as a whole of school approach has been associated with greater implementation of programmes in schools (Colabianchi *et al.*, 2015) and can lead to increased engagement with school communities for sustainable change (WHO, 2017a).

Teachers suggested a number of key enablers and or barriers to them understanding and implementing HL in their practice. First, teachers suggested that the programme fostered the development of shared language. Other publications assessing the impact of HealthLit4Kids support this suggestion, as well as reporting the development of shared language for other members of the school community (Nash *et al.*, 2019, 2020, 2021a; Elmer *et al.*, 2021). This is important, given collaboration is reliant on effective communication (Joyce and Deana, 2013). Second, teachers highlighted the importance of supportive leadership and department support to programme implementation. Consistent with other research on the initiative, the programme has been found to promote supportive leadership, however, additional support could help to further mitigate barriers (Nash *et al.*, 2019; Elmer *et al.*, 2021). Third, teachers reported their plans to use their newfound knowledge from the workshops to communicate with parents. Parent interviews demonstrated that HealthLit4Kids led to the development of HL of parents, and that parents reported valuing the programme (Nash *et al.*, 2020). This is encouraging, given parental engagement is important to ensure student HL asset development can be reinforced in the home as well as the school environment.

### Resources

Teacher evaluations reported that further resources such as additional PD, information available for parents to access, support time, access to health professionals (e.g. school psychologist, social worker), teaching resources (outlining practical methods of how to embed HL into lessons), as well as further financial aid are needed to help support HL implementation in schools. Multiple studies examining teacher experience of the initiative have reflected this finding (Nash *et al.*, 2019, 2021a; Elmer *et al.*, 2021). This implies that whilst the HealthLit4Kids programme aims to address the gap in resources (Nash *et al.*, 2020) and has demonstrated positive impact (Nash *et al.*, 2019, 2021a; Elmer *et al.*, 2021), barriers to implementing the initiative still exist.

Time was one of the most predominantly discussed barriers in the teacher evaluations. Some teachers reported time as the only barrier to implementing HL into their lessons. Time has been reported elsewhere as a major barrier to applying PD across sectors (i.e. healthcare settings) (Hemmington, 2000; Ikenwilo and Skåtun, 2014) and in educational settings (Duignan *et al.*, 2016; Brigandi *et al.*, 2019; Schwartz *et al.*, 2019). It has also been a finding of a number of HealthLit4Kids project reports (Nash *et al.*, 2019, 2021a; Elmer *et al.*, 2021). Workshop evaluations suggested that teachers faced time barriers due to too many competing curricular demands.

Some teachers suggested that integration of HL into other learning areas could help to mitigate the barrier of time. An integrated, rather than siloed approach to teaching and learning connects different areas of study simultaneously (Moss *et al.*, 2019; Minić and Jovanović, 2020) and facilitates meaningful learning experiences that are contextually appropriate to students (Moss *et al.*, 2019; Minić and Jovanović, 2020). This is particularly relevant to education that develops HL, given the contextual nature of the asset.

Workshop evaluations revealed that the PD was useful as it allowed teachers time to focus on HL. This implies that they were engaged with the PD, which could further explain why teachers suggested an improvement in their professional knowledge. Time is often a perceived barrier to implementing PD, but it can be reduced with increased understanding (Brownell and Tanner, 2012). Consequently, by improving teacher’s understanding of HL (PK), as well as understanding of how to teach HL (PCK), it could mitigate their perception of this barrier.

### Strengths and limitations

Strengths of this study include co-designed workshops (Steen *et al.*, 2011), consistent facilitation by one facilitator at all three schools and multi-site delivery providing greater confidence in the generalizability of the results (Flynn, 2009). The convergent and sequential design of the study is a strength as one design can help to overcome the limitations of another (Creswell, 2013; Greenhalgh *et al.*, 2016; O’Leary, 2017). Finally, this study is original research in an area that has not previously been explored, therefore, it contributes new insight in the HL field.

Limitations of this study include convenience sampling (Liamputtong *et al.*, 2016) and incomplete data from the five participating schools. This limited the analysis to three schools reducing statistical power of quantitative results and the representativeness of the sample (Hyun, 2013). To mitigate this concern, schools from varying demographics were chosen.

Currently there are no validated tools for evaluating HL programmes, the instrument we used was not reliability or validity tested. If used in the future it may require adjusting, given participant response fatigue existed (Egleston *et al.*, 2011; O’Reilly-Shah, 2017). Further, whilst a self-assessment tool (such as the questionnaire used in this research) can be used as an indicator of HL knowledge, using a validated HL measure could help to strengthen the conclusions surrounding improved HL knowledge of teachers. Finally, we acknowledge that self-reported questionnaires can be subject to social desirability response bias (Larson, 2019).

## CONCLUSION

Findings from this study suggest that teachers perceived that their involvement with HealthLit4Kids improved their confidence and competence to implement HL into their lessons. As a result, it improved student access to education that develops HL*.* Teachers highlighted the importance of collaborative teaching strategies to optimize planning and learning opportunities. Additional resources (i.e. further HL PD, adaptable teacher resources) may help to support teacher understanding of HL and consequent implementation into practice. This could help to mitigate reported barriers (e.g. time) to implementation, thereby, resulting in greater incorporation of HL into curriculum. Given that HL has the potential to impact student’s education and health outcomes and children have a right to education that develops HL (2018), this research is critically important to the field.

## SUPPLEMENTARU MATERIAL

Supplementary material is available at *Health Promotion International* online.

## Supplementary Material

daac053_Supplementary_Data
