# Supplementary material for: HealthLit4Kids: teacher experiences of health literacy professional development in an Australian primary school setting
Source: Health Promot Int. 2022 May 11;38(3):daac053. doi: 10.1093/heapro/daac053 (PMC10269120; doi:10.1093/heapro/daac053)
Supplement: daac053_Supplementary_Data [file daac053_supplementary_data.zip › daac053_Supplementary_Data/Supplementary Table 1new.docx]

| **Supplementary Table 1.** School Demographics, Workshop Dates, and Participants | | | | | | | | |
| --- | --- | --- | --- | --- | --- | --- | --- | --- |
| **School** | **Date of**  **Workshops** | **Location** | **TSCA * Accredited** | **MWEW** School** | **SEIFA^†‡^ Decile (within Australia)** | **Number of Students** | **Number of Teaching Staff** | **Number of Teachers that Responded**  **W1, W2, W3** |
|  |  |  |  |  |  |  |  |  |
| 1 | **1)**24/05/17  **2)**28/06/17  **3)**22/11/2017 | Inner Regional | Yes | Yes | 8 | 297 | 18 | 10, 16, 11 |
| 2 | **1)** 29/05/18  **2)** 19/06/18  **3)** 06/11/18 | Inner Regional | Yes | Yes | 2 | 289 | 27 | 22, 11, 18 |
| 3 | **1)** 15/05/18  **2)** 12/06/18  **3)** 20/11/18 | Outer Regional | No | Yes | 2 | 366 | 29 | 10, 9, 6 |

SEIFA - Socio-Economic Indexes for Areas. † SEIFA score is an indicator of the relative socio-economic advantage or disadvantage in an area on a scale with a mean of 1000 and standard deviation of 100. ‡ SEIFA decile is an indicator of the areas SEIFA distribution in ten equal groups (where 1 is the lowest score and 10 is the highest). *Tasmanian School Canteen Association accreditation indicates if schools are gold, silver, or bronze based on canteen management and promotion of healthy eating. **Move Well Eat Well members schools can access health eating resources and support from the Department of Health and Human Services Tasmania.
