# Supplementary material for: HealthLit4Kids: teacher experiences of health literacy professional development in an Australian primary school setting
Source: Health Promot Int. 2022 May 11;38(3):daac053. doi: 10.1093/heapro/daac053 (PMC10269120; doi:10.1093/heapro/daac053)
Supplement: daac053_Supplementary_Data [file daac053_supplementary_data.zip › daac053_Supplementary_Data/Supplementary Table 2new.docx]

| Supplementary Table 2. *HelathLit4Kids Prior Research Findings in Support of Teacher Reflections* | | | | | | | |
| --- | --- | --- | --- | --- | --- | --- | --- |
| **Parent Theme** | **Sub-theme** | **Paper^[[1]](#footnote-1)^** | | | | | |
|  |  | **1** | **2** | **3** | **4** | **5** | **6** |
| Teacher Development | The professional development led to an improved **understanding** of the term and concept of health literacy. | ✓ |  | ✓ | ✓ |  | ✓ |
|  | The professional development led teachers to a greater awareness of how to **implement health literacy** into their **classroom practice.** |  |  | ✓ |  |  | ✓ |
|  | Teachers understanding of how to implement health literacy into the curriculum **developed over the three workshops.** |  |  | ✓ | ✓ |  | ✓ |
|  | The professional development led to an improved **confidence** in relation to health and/or health literacy. |  |  |  |  |  | ✓ |
|  | **Reflection** was a useful element of the health literacy professional development. |  |  |  |  |  |  |
|  | At the conclusion of the health literacy professional development teachers reported **valuing health literacy**. |  |  | ✓ |  |  |  |
| Collaborative Practice | The workshops led to an **improved collective understanding** of health literacy and its relevance to the **whole of school.** | ✓ |  |  | ✓ | ✓ |  |
|  | **Discussions and the opportunity to work collaboratively** were useful elements of the professional development/ helped to improve health literacy understanding. | ✓ |  |  | ✓ |  | ✓ |
|  | A **whole of school** approach to a health literacy initiative is critical for it to be effective. | ✓ | ✓ |  | ✓ | ✓ | ✓ |
|  | A strength of the professional development was that it employed a **co-design.** | ✓ | ✓ |  | ✓ | ✓ | ✓ |
|  | Support from **senior management** is key to health literacy program effectiveness. |  |  |  | ✓ |  |  |
|  | **Parental/family involvement is important** when implementing an initiative. | ✓ |  |  | ✓ | ✓ |  |
|  | Developing **shared language** is important when implementing an initiative. | ✓ |  |  | ✓ | ✓ | ✓ |
| Resources | **Time** was one of the greatest perceived **barriers** to teachers implementing health literacy into their lessons. |  |  | ✓ | ✓ | ✓ | ✓ |
|  | The professional development was useful as it **allowed teachers time to focus on health literacy.** |  |  |  |  |  |  |
|  | **Further resources** would help to support health literacy implementation in schools. |  |  | ✓ | ✓ | ✓ | ✓ |

1. **1** = (Nash et al., 2020) **2** = (Nash et al., 2018) **3.** (Cruickshank et al., 2020) 4. (Elmer et al., 2020) 5. (Nash et al., 2019) **6.** (Nash et al, 2021a) [↑](#footnote-ref-1)
