# Supplementary material for: HealthLit4Kids: teacher experiences of health literacy professional development in an Australian primary school setting
Source: Health Promot Int. 2022 May 11;38(3):daac053. doi: 10.1093/heapro/daac053 (PMC10269120; doi:10.1093/heapro/daac053)
Supplement: daac053_Supplementary_Data [file daac053_supplementary_data.zip › daac053_Supplementary_Data/Supplementary Table 3new.docx]

| Supplementary Table 3. *Qualitative Data Analysis. Thematic Analysis* | | | | | | | |  |
| --- | --- | --- | --- | --- | --- | --- | --- | --- |
| **Parent Theme** | **Sub-Theme** | **Illustrative Quotes** | | | **School** | | |  |
|  |  |  |  |  | **A** | **B** | **C** | |
|  |  |  | | |  |  |  | |
| Teacher Development | The professional development led to an improved **understanding** of the term and concept of health literacy. | “I have a **clearer understanding** of Health Literacy after the session” (Teacher 4, Workshop 1).  The workshop led to a “**greater understanding of health literacy** and its relevancy to school” (Teacher 7, Workshop 2).  “**Consolidated my understanding** of health literacy…” (Teacher 1, Workshop 3).  “In the beginning I only had an ‘educated guess’ as to what Health Literacy was. **Now I’m not ‘guessing’**” (Teacher 11, Workshop 3).  “I now have a **greater understanding** of what it all means” (Teacher 2, Workshop 3). | | | ✓ | ✓ | ✓ | |
|  | The professional development led to an improved **confidence** in relation to health and/or health literacy. | “Much more **comfortable** and clear of definition after this meeting” (Teacher 14, Workshop 2).  “I thought I knew what health literacy meant but wasn’t quite sure. After attending the workshop, I am now **confident** that I understand the term” (Teacher 2, Workshop 1).  “I now feel **more comfortable** when using/understanding the jargon involved in this study” (Teacher 2, Workshop 3).  “I’m now able to assist parents with information. Highlight ‘health’ related areas. Plus **feel confident teaching health**” (Teacher 11, Workshop 3). | | | ✓ |  |  | |
|  | The professional development led teachers to a greater awareness of how to **implement health literacy** into their **classroom practice.** | **“**Through opportunities to discuss issues: stimulated ideas, **provoked thoughts of how programs/changes in procedures could be implemented**” (Teacher 15, Workshop 2).  “**Now aware** of the breadth of areas health literacy covers and the **number of programs and strategies** that already exist at *School A* that can be mapped back to health literacy” (Teacher 16, Workshop 2).  “Consolidated my understanding of health literacy- and **how I can apply it”** (Teacher 1, Workshop 3).  **“**I hadn’t thought a lot about the ‘big picture’ in terms of Health Literacy in my present position at this school. **I’m now more aware of what it might mean for us”** (Teacher 2, Workshop 1).  “Gave an excellent understanding of all the elements involved in Health Literacy and **how they apply to all areas of the school community**” (Teacher 3, Workshop 1). | | | ✓ | ✓ | ✓ | |
|  | Teachers understanding of how to implement health literacy into the curriculum **developed over the three workshops**. | **Workshop 1** | **Workshop 2** | **Workshop 3** | ✓ |  | ✓ | |
|  |  | “Yes, but still needs more understanding through ongoing workshops to make sure everyone understands well enough to take on board and move forward” (Teacher 5, Workshop 1).  “A clear definition but not yet knowledge and understanding to enable application” (Teacher 7, Workshop 1).  “The presenters were excellent and opened up lots of discussion around health literacy. I am still unsure how to apply this in an early childhood setting as I feel the families need to be involved” (Teacher 9, Workshop 1).  “I’m gaining an understanding of the elements (of health literacy)- actually I think I might have known more than I realised” (Teacher 4, Workshop 1).  “Still needs more understanding through ongoing workshops to make sure everyone understands well enough to take on board and move forward” (Teacher 5, Workshop 1).  “Still unclear as to how this will be applied in classroom” (Teacher 8, Workshop 1). | “Much more comfortable and clearer of definition after this meeting” (Teacher 14, Workshop 2).  “Helped clarify understanding, change my own concept of health literacy like moderation of all our thoughts” (Teacher 5, Workshop 2).  “[This workshop brought] greater understanding of health literacy and its relevancy to school. Still in need of school plan and practical resources to use” (Teacher 7, Workshop 2).  “Continued analysis of our current and past practices and experiences, shared in an environment of trust, has brought more understandings” (Teacher 11, Workshop 2).  “Good to recap and ensure understanding” (Teacher 13, Workshop 2).  “Now aware of the breadth of areas health literacy covers and the number of programs and strategies that already exist at school A that can be mapped back to health literacy” (Teacher 16, Workshop 2). | “Consolidated my understanding of health literacy- and how I can apply it” (Teacher 1, Workshop 3).  “I now feel more comfortable when using/understanding the jargon involved in this study” (Teacher 2, Workshop 3).  “The understanding of health literacy became very clear as time evolved” (Teacher 7, Workshop 3).  “In the beginning I only had an ‘educated guess’ as to what health literacy was. Now I’m not ‘guessing’” (Teacher 11, Workshop 3).  “Through team planning and reflection I’ve grown to see how health literacy applies to us [teachers]” (Teacher 17, Workshop 3).  “I’m now able to assist parents with information. Highlight ‘health’ related areas. Plus feel confident teaching health” (Teacher 7, Workshop 3). |  |  |  |  |
|  | **Reflection** was a useful element of the health literacy professional development. | “Through team planning and **reflection** I’ve grown to see how health literacy applies to us [teachers]” (Teacher 1, Workshop 3).  *What was useful about the workshop?*  “Increased my understanding and gave planning/reflection time” (Teacher 11, Workshop 3).  “Time to talk, discuss, reflect, sort out jargon etc.” (Teacher 2, Workshop 3).  “Whole school involvement, reflection and common approach, sharing and celebration of artefacts across the school” (Teacher 5, Workshop 3).  “A chance to discuss and reflect upon the varied school achievements this year” (Teacher 6, Workshop 3). | | | ✓ | ✓ | ✓ | |
|  | At the conclusion of the health literacy professional development teachers reported **valuing health literacy**. | “I definitely agree with the health literacy focus areas for our school. Because of the changing nature of society. Important to work with school as a community not just in isolation” (Teacher 1, Workshop 1).  “Health literacy focus is very important” (Teacher 6, Workshop 1).  “It’s [health literacy] is critical” (Teacher 5, Workshop 2).  “Beginning the conversation of what ‘Health literacy’ is the most critical part of this project- it is such a neglected area of curriculum” (Teacher 15, Workshop 1).  “[The health literacy professional development was useful as it] made me think about health literacy” (Teacher 3, Workshop 1).  “Always good to switch peoples thinking back to the importance/relevance of health and help develop understandings” (Teacher 5, Workshop 1). | | | ✓ | ✓ | ✓ | |
| Collaborative Practice | The workshops led to an **improved collective understanding** of health literacy and its relevance to the **whole of school.** | “I **hadn’t thought a lot about the ‘big picture’** in terms of health literacy in my present position at this school. I’m now **more aware** of what it might mean for us” (Teacher 2, Workshop 1).  “Gave an excellent understanding of all the elements involved in health literacy and how they apply to all areas of the **school community**” (Teacher 3, Workshop 1).  “We are starting to discuss ideas for our artefacts and found topics that will help shine a spotlight on **elements of health literacy in our school** e.g. mindfulness, healthy food and drink choices, movement for health” (Teacher 1, Workshop 2).  “The workshops have enabled us to undertake conversations at a **whole of teaching staff level** that have supported us in developing our **collective understanding** of the elements that influence the health literacy of the school environment” (Teacher 16, Workshop 2).  “**School wide focus as well** as class focus for health literacy was clearly outlined” (Teacher 6, Workshop 3). | | | ✓ |  | ✓ | |
|  | **Discussions and the opportunity to work collaboratively** were useful elements of the professional development/ helped to improve health literacy understanding. | “The workshops have enabled us to undertake conversations at a whole of teaching staff level that have supported us in developing our collective understanding of the elements that influence the health literacy of the school environment” (Teacher 16, Workshop 2).  “Through team planning and reflection I’ve grown to see how health literacy applies to us [teachers]” (Teacher 1, Workshop 3).  “Peer discussions have supported my understandings” (Teacher 4, Workshop 3).  “Discussion with all staff is interesting and worthwhile” (Teacher 6, Workshop 2).  “Through opportunities to discuss issues: stimulated ideas, provoked thoughts of how programs/changes in procedures could be implemented” (Teacher 15, Workshop 2). | | | ✓ | ✓ | ✓ | |
|  | A **whole of school** approach to a health literacy initiative is critical for it to be effective. | “Knowing the whole school was engaged in health literacy allowed open/broader discussion” (Teacher 8, Workshop 3).  “Setting up standards in our school would be great as long as we’re ALL on board” (Teacher 2, Workshop 2).  “The school environment has had a wonderful impact with whole school collaboration” (Teacher 3, Workshop 3).  “Important to work with school as a community not just in isolation” (Teacher 1, Workshop 1).  “Teachers, children, parents need to understand what/where/why and we all need to work together to improve all of our health literacy” (Teacher 2, Workshop 1). | | | ✓ | ✓ | ✓ | |
|  | A strength of the professional development was that it employed a **co-design.** | “Through team planning and reflection I’ve grown to see how health literacy applies to us [teachers]” (Teacher 1, Workshop 3).  “Certainly, planning and working with staff to develop learning and artefacts gave depth to our work with students” (Teacher 2, Workshop 1).  “Good to be able to target areas identified by staff as relevant to our school community” (Teacher 5, Workshop 1).  “Understanding more about health literacy and working together to plan were the most useful parts of the workshop” (Teacher 5, Workshop 1). | | | ✓ | ✓ | ✓ | |
|  | Support from **senior management** is key to health literacy program effectiveness. | “Needs to come from top down and be advertised/sold to school/greater community” (Teacher 2, Workshop 2).  “Community involvement, department support, leadership on board [could help to remove barriers]” (Teacher 6, Workshop 1).  “Education Department needs to be on board” (Teacher 2, Workshop 2).  “ [Support from] education Department. Parents putting pressure on politicians” (Teacher 12, Workshop 2). | | | ✓ |  |  | |
|  | **Parental/family involvement is important** when implementing an initiative. | “Educate parents! And teachers and children. Ask GPs to educate individuals as they come into their surgeries” (Teacher 2, Workshop 1).  “Parents need to come on board and stop sending packaged/unhealthy foods in lunch boxes. They aren’t familiar or educated about unhealthy/healthy foods and/or they don’t’ care!” (Teacher 2, Workshop 1).  “Parent participation [is key in reducing barriers]” (Teacher 4, Workshop 1).  “Accessing information/resources to help inform parents [is key in reducing barriers]” (Teacher 7, Workshop 2).  “Accessing new info/resources e.g., medical in order to be better support families – e.g. pamphlets; Funding personnel available & timeframes e.g., school psych, social worker [is key in reducing barriers]” (Teacher 1, Workshop 3). | | | ✓ | ✓ | ✓ | |
|  | Developing **shared language** is important when implementing an initiative. | “I now feel more comfortable when using/understanding the jargon involved in this study” (Teacher 2, Workshop 3).  “In the beginning I only had an ‘educated guess’ as to what health literacy was. Now I’m not ‘guessing’” (Teacher 11, Workshop 3).  “Lots of new terminology and the possibility of re-igniting “health” education in a new way” (Teacher 9, Workshop 1).  “Time to talk, discuss, reflect, sort out jargon etc.,” were the most beneficial elements to the workshop (Teacher 6, Workshop 3). | | | ✓ |  | ✓ | |
| Resources | **Time** was one of the greatest perceived **barriers** to teachers implementing health literacy into their lessons. | *Are there any barriers to you using this information?*  “**Time** and further planning” (Teacher 5, Workshop 1).  “Time factor” (Teacher 10, Workshop 2).  “Incorporating into full curriculum” (Teacher 9, Workshop 2).  *“*Time*”* (Teacher 23, Workshop 2).  “We have a very crowded curriculum and lots of extra stuff, sports, daily PE, assemblies” (Teacher 9, Workshop 1). | | | ✓ | ✓ | ✓ | |
|  | The professional development was useful as it **allowed teachers time to focus on health literacy.** | *What was useful about the workshops?*  “Supported **time** and resources to involve support staff including teacher assistants and admin staff in a whole of school approach” (Teacher 16, Workshop 2).  “Time to discuss these important core issues with independent facilitators [was the most useful part of the workshop]” (Teacher 9, Workshop 2).  “Increased my understanding and gave **planning/reflection time**” (Teacher 1, Workshop 3).  “**Time to talk**, discuss, reflect, sort out jargon etc.” (Teacher 2, Workshop 3). | | | ✓ | ✓ | ✓ | |
|  | **Further resources** are would help to support health literacy implementation in schools. | *What would help to reduce barriers to implementing health literacy?*  “Resources- school follow up and department support” (Teacher 6, Workshop 1).  “Education department needs to find $$” (Teacher 2, Workshop 2).  “Yes, accessing information/resources to help inform parents” (Teacher 7, Workshop 2).  “Supported time and **resources** to involve support staff including teacher assistants and admin staff in a whole of school approach” (Teacher 16, Workshop 2).  “Accessing new info/resources e.g., medical in order to be better support families – e.g. pamphlets; Funding personnel available & timeframes e.g., school psych, social worker” (Teacher 1, Workshop 3).  “Access to info and resources [would help remove the barriers]; Funding & personnel” (Teacher 11, Workshop 3). | | | ✓ | ✓ | ✓ | |
