# Supplementary figures and images for: HealthLit4Kids: teacher experiences of health literacy professional development in an Australian primary school setting
Source: Health Promot Int. 2022 May 11;38(3):daac053. doi: 10.1093/heapro/daac053 (PMC10269120; doi:10.1093/heapro/daac053)

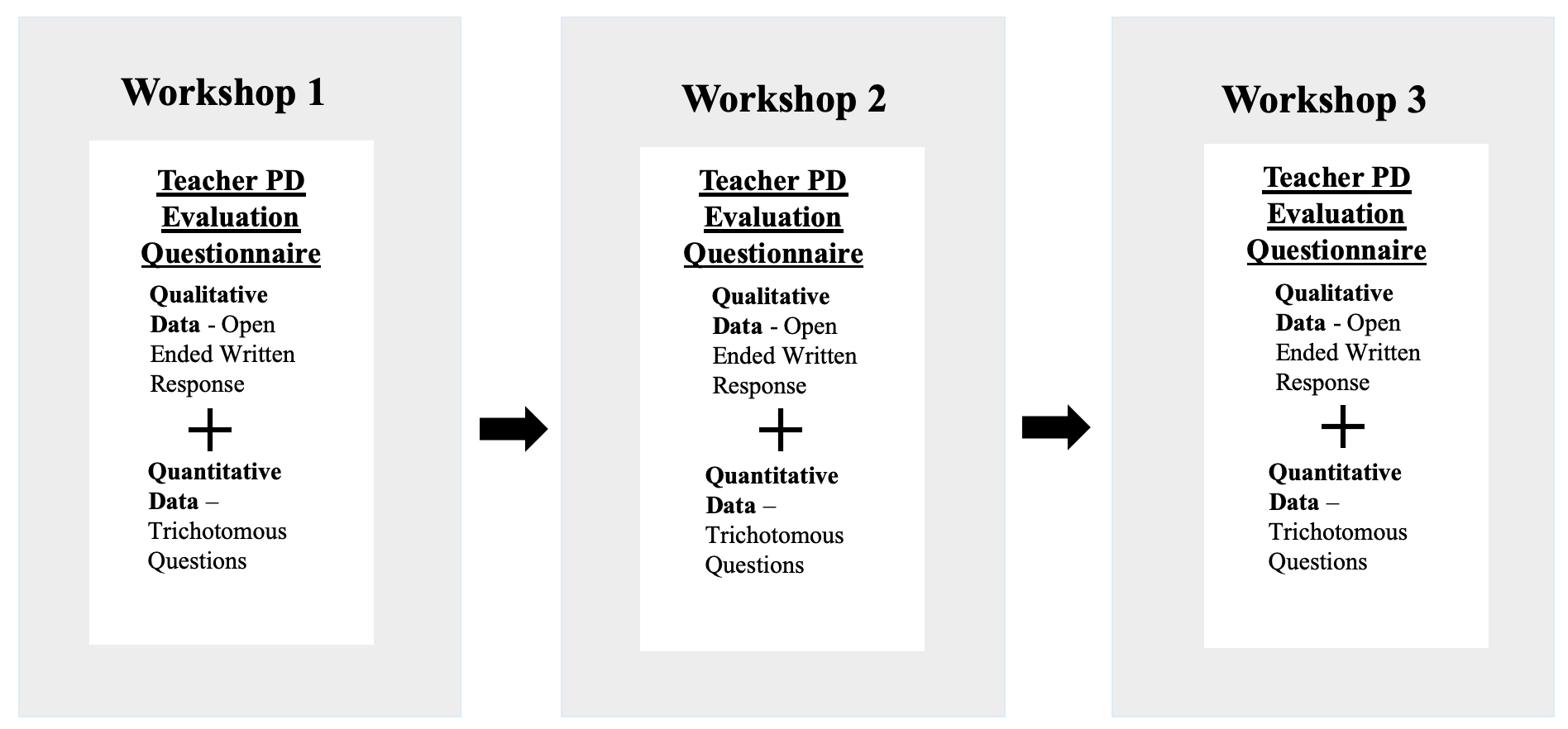

Supplement: daac053_Supplementary_Data [file daac053_supplementary_data.zip › daac053_Supplementary_Data/Supplementary Figure 1 - Design of Study- Multi-Phased Mixed Methodsnew.jpg]

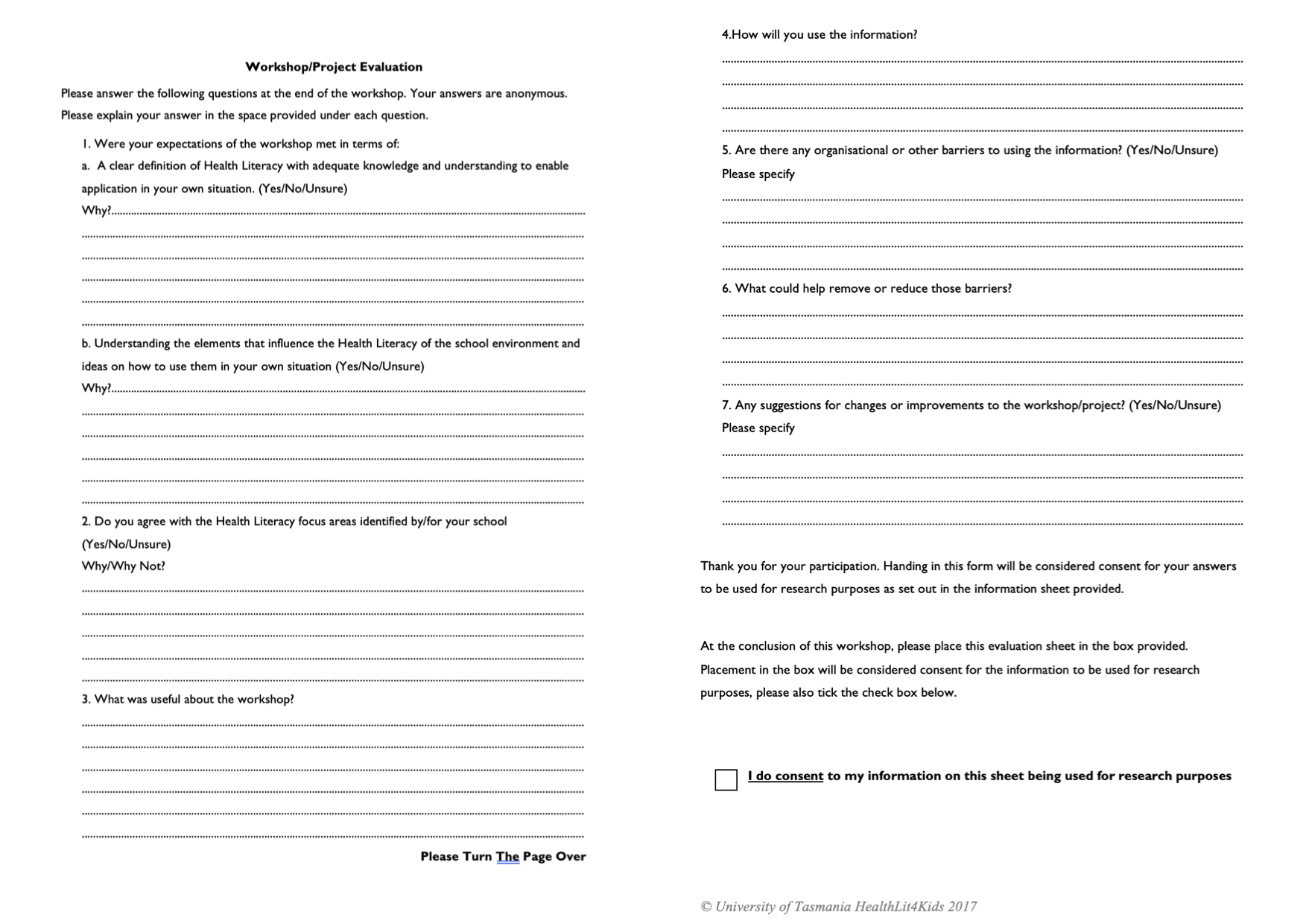

Supplement: daac053_Supplementary_Data [file daac053_supplementary_data.zip › daac053_Supplementary_Data/Supplementary Figure 2 - Questionnairenew.jpg]
